# Synergistic interaction between trazodone and gabapentin in rodent models of neuropathic pain

**DOI:** 10.1371/journal.pone.0244649

**Published:** 2021-01-04

**Authors:** Beatrice Garrone, Anna di Matteo, Alessandro Amato, Luana Pistillo, Lucia Durando, Claudio Milanese, Francesco Paolo Di Giorgio, Serena Tongiani

**Affiliations:** Angelini Pharma S.p.A., Rome, Italy; University of Arizona College of Medicine, UNITED STATES

## Abstract

Neuropathic pain is a chronic debilitating condition caused by injury or disease of the nerves of the somatosensory system. Although several therapeutic approaches are recommended, none has emerged as an optimal treatment leaving a need for developing more effective therapies. Given the small number of approved drugs and their limited clinical efficacy, combining drugs with different mechanisms of action is frequently used to yield greater efficacy. We demonstrate that the combination of trazodone, a multifunctional drug for the treatment of major depressive disorders, and gabapentin, a GABA analogue approved for neuropathic pain relief, results in a synergistic antinociceptive effect in the mice writhing test. To explore the potential relevance of this finding in chronic neuropathic pain, pharmacodynamic interactions between low doses of trazodone (0.3 mg/kg) and gabapentin (3 mg/kg) were evaluated in the chronic constriction injury (CCI) rat model, measuring the effects of the two drugs both on evoked and spontaneous nociception and on general well being components. Two innate behaviors, burrowing and nest building, were used to assess these aspects. Besides exerting a significant antinociceptive effect on hyperalgesia and on spontaneous pain, combined inactive doses of trazodone and gabapentin restored in CCI rats innate behaviors that are strongly reduced or even abolished during persistent nociception, suggesting that the combination may have an impact also on pain components different from somatosensory perception. Our results support the development of a trazodone and gabapentin low doses combination product for optimal multimodal analgesia treatment.

## Introduction

Neuropathic pain is a chronic and debilitating condition caused by injury or disease of the nerves of the somatosensory system [1]. Because of the heterogeneity of its aetiologies, symptoms and underlying mechanisms [2], current pharmacological treatments encompass different drug classes. Given the small number of approved drugs and their limited clinical efficacy, at least 45% of patients with neuropathic pain concurrently receive two or more drugs to treat their condition [3]. In fact, combining drugs with different pharmacological mechanisms of action may yield greater efficacy and a greater chance of modulating multiple pain mechanisms [4].

Pain comprises two main components: the somatosensory one, responsible for determining the location and intensity of pain, and an affective component, which consists in a complex cognitive and emotional experience which depends also on the psychological state of the individual [5,6]. Although the link between emotional state and pain perception has been clearly confirmed by several controlled studies both in healthy volunteers and in patients [6], the impaired cognitive and emotional states that are often concomitant in patients with chronic pain are not properly addressed with current therapeutic approaches. As a consequence, new drugs for the treatment of neuropathic pain that can also affect these aspects are needed.

Gabapentin is an anticonvulsant drug originally registered for the treatment of epilepsy and recently approved for some neuropathic pain forms. Gabapentin selective inhibition of the α_2_-δ subunits of voltage-sensitive calcium channels (VSCC) [7] is implicated in reducing allodynia because it causes reduction of neuronal excitability and modulation of neurotransmitter release. However, its use is affected by dose-limiting side effects requiring prolonged dose titration.

With the aim of targeting the impaired affective components of painful conditions, antidepressant drugs are commonly used in the clinical setting for the treatment of chronic pain [8]. Combinations of antidepressants with gabapentin showed superiority to gabapentin monotherapy both in preclinical [9,10] and clinical settings [11–13]. Trazodone is a multifunctional drug approved worldwide for the treatment of major depression with a mild adverse effect profile (sedation). It is a blocker of the post-synaptic serotonin (5-HT) receptors 5-HT_2A_ and 5-HT_2C_ and inhibitor of presynaptic 5-HT reuptake transporters [14]. Trazodone, tested in the chronic constriction injury (CCI) rat model of chronic pain [15], showed a dose-dependent analgesic effect on thermal hyperalgesia [16].

Hence, because the antinociceptive effects of gabapentin and trazodone are mediated by different mechanisms, we hypothesized that the drugs could synergistically interact when concomitantly administered. Using an acute pain model, the writhing test, we confirmed such assumption through isobolographic analysis. We then evaluated pharmacodynamic interactions between low doses of trazodone and gabapentin in the rat CCI model, characterizing their effects both on evoked and spontaneous nociception and on behaviors reflecting general animal well being. To this specific purpose, the burrowing test and the nest construction assays were performed. They are naturally occurring behaviours in both rats and mice, and they have been used as an indicator of distress and suffering in several disease models [17–19].

Besides exerting a significant antinociceptive effect on hyperalgesia and on spontaneous pain, the combination of inactive doses of trazodone and gabapentin restored rat innate behaviors that are strongly reduced or even abolished during persistent nociception, suggesting that the combination may have an impact also on aspects of chronic pain different from the somatosensory components.

## Materials and methods

### Animals

Experiments were performed on male CD1 mice weighing 20–25 g and male CD rats weighing 200–225 g (Charles River, Italy). The animals were housed in groups of 5 mice or 4 rats in solid bottom polypropylene cages. Room temperature and relative humidity were set at 22±2°C and 55±15%, respectively, and the lighting was controlled on a cycle of 12 hour light and 12 hour darkness. Food and water were freely available, except during the experimental procedure. All experimental sessions were performed between 9:00 am and 1:00 pm to avoid diurnal variation in the behavioural tests. Each pain test was performed by an experimenter blinded to the treatments. Animals were grouped with homogeneous baseline threshold.

The experiments were carried out in accordance with the guidelines established by the the European Communities Council Directive (Directive 2010/63/EU of 22 September 2010) and approved by the National Council on Animal Care of the Italian Ministry of Health (Authorization n. 59/2013-B). All efforts were made to minimize animal suffering and to use the minimal number of animals required to produce reliable results.

### Drugs

Trazodone (Angelini SpA, Italy) and gabapentin (Sigma-Aldrich, St. Louis, MO, USA) were used. These drugs and their combination were dissolved in 0.9% NaCl and injected by gavage (trazodone) or intraperitoneally (gabapentin). Acetic acid was purchased from Sigma-Aldrich, dissolved in 0.9% NaCl and injected and injected intraperitoneally.

### Acetic acid-induced writhing test in mice

Mice were intraperitoneally injected with 16 μl/g body weight of 0.7% (v:v) acetic acid, and the number of writhes was counted during a 10 min period, starting 5 min after administration of acetic acid solution [20]. A writhe was defined as a contraction of the abdominal muscles accompanied by an elongation of the body and extension of the hindlimbs. Drugs were administered alone or in combination (or the vehicle in the control group) 1 h before the acetic acid injection. The results are expressed as the number of writhes. The values of ED_50_ (the drug dose producing 50% of a maximal effect) were calculated by linear regression analysis from trazodone (0.1–3 mg/kg, p.o.) and gabapentin (1–100 mg/kg, i.p.) dose-response curves. Doses were administered in 10 ml/kg body weight.

### Isobolographic analysis

The interaction between trazodone and gabapentin was evaluated by co-administration of fixed proportions of each drug and performing an isobolographic analysis. Briefly, according to the method described by Tallarida [21], we plotted the ED_50_ value of each drug alone on the x- and y-axes of the isobologram. Then, the line joining the x- and y-axes corresponds to the theoretical additive line (isobole). The point represented in the isobole line is the theoretical additive point of the combined treatment.

In the next step, trazodone and gabapentin were co-administered at fixed-dose fractions of the ED_50_ (1/2, 1/4, 1/8 or 1/16) of each drug. For the drug mixture, experimental ED_50_ (ED_50 mix_) was determined by linear regression analysis of the dose-response curve and compared with a theoretical additive ED_50_ (ED_50 add_) obtained from the calculation:

$${\text{ED}}_{50\;\text{add}}=\;\text{f}\;\times {\;\text{ED}}_{50\;\text{TRAZODONE}}+\;(1-\text{f})\;\times {\;\text{ED}}_{50\;\text{GABAPENTIN}}$$

where f denotes a fraction of the corresponding ED_50_ in the mixture (in our study, f = 0.5).

If the ED_50 mix_ falls below or above the isobole, synergy or antagonism, respectively, is said to occur between the combined drugs.

Additionally, to describe the magnitude of the interaction, the interaction index (γ) was calculated according to the following formula:

$$\gamma ={\;\text{ED}}_{50\;\text{TRAZODONE}\;\text{COMBINED}\;\text{WITH}\;\text{GABAPENTIN}}/{\text{ED}}_{50\;\text{TRAZODONE}\;\text{GIVEN}\;\text{ALONE}}+{\;\text{ED}}_{50\;\text{GABAPENTIN}\;\text{COMBINED}\;\text{WITH}\;\text{TRAZODONE}}/{\text{ED}}_{50\;\text{GABAPENTIN}\;\text{GIVEN}\;\text{ALONE}}$$

The interaction index γ is a quantitative marker for the drug combination that indicates the changed potency of the combination. Values near 1 indicate additive interaction; values >1 imply antagonism, whereas values <1 indicate a synergistic interaction.

### Chronic constriction injury in rats

#### Surgery and drug treatment

A neuropathic pain model of chronic constriction injury (CCI) described by Bennet and Xie [22] was applied. The day before surgery, rats were individually housed. Under 1.5% isofluorane anesthesia, the sciatic nerve of the left hind paw was exposed at the level of the middle of the thigh by dissection through biceps femoris. The nerve was freed of adhering tissue, and three loose ligatures, the first in the middle of the section exposed, were tied around it with approximately 3 mm spacing.

In order to test for a synergistic effect, we administered a combination of trazodone and gabapentin ineffective doses (trazodone 0.3 mg/kg, p.o. and gabapentin 3 mg/kg, i.p.) and examined the effects on mechanical hyperalgesia, weight bearing deficit, burrowing and nest construction behaviour. Doses were administered in 10 ml/kg body weight. Naive rats were used as positive control, while ligated rats receiving vehicle were used as negative control.

### Mechanical hyperalgesia

To assess the mechanical hyperalgesia, paw withdrawal thresholds, measured in grams (g), were determined using an Analgesymeter (Ugo Basile, Varese, Italy), according to the method described by Randall and Selitto [23]. Increasing pressure (20 g/s up to 500 g max.) was applied until animal struggled or squealed.

Mechanical hyperalgesia was determined before the surgery (baseline), 3, 7 or 14 days following sciatic nerve ligation before the acute drugs administration (pre-drug) and 1 h after (post-drug). The percentage reversal (% maximal possible effect, MPE) ± SEM of hyperalgesia, was calculated as:

$$\frac{\text{Post-drug}\;\text{threshold}\;-\;\text{pre-drug}\;\text{threshold}}{\text{Baseline}\;\text{threshold}\;-\;\text{pre-drug}\;\text{threshold}}\;\times \;100$$

### Weight bearing

Static weight bearing is useful for measuring spontaneous pain [24] and weight bearing changes were measured using an automatic incapacitance tester (2B, 2biological instruments, Varese, Italy), a device that measures the weight distributed to each hind paw individually. The value reported for each animal was the mean of 3 consecutive measurements. In the absence of hind limb injury, rats applied an equal weight on both hind limbs, indicating a postural equilibrium, whereas an unequal distribution of the weight on hind limbs indicated a monolateral decreased pain threshold.

On days 3, 7 and 14 post-surgery, weight bearing deficit was measured before the drugs administration and 1 h after. The results (mean ± SEM) are expressed as the difference between the weight applied to the limb contralateral to the injury and the weight applied to the ipsilateral one, reading in grams (g).

### Burrowing test

The burrowing test is based on the typical behaviour of spontaneously displacing items from tubes within the animal’s home cage. In this study, we filled a plastic tube (34 cm in length and 10 cm in diameter) with 1.5 kg of gravel (composed of stones 2–4 mm in diameter). The open end of the tube was elevated 3 cm from the floor of the cage, as reported in Deacon [17].

All rats received four training sessions prior to the surgery and the burrowing test. For training, rats were placed in cages with empty burrowing tubes (60 min on day 1). On days 2–4, animals spent 30 min in an empty cage, after which the gravel filled tube was introduced for a 60 min test session. Rats were placed into cages in pairs for training sessions on days 1 and 2 and on their own for training sessions on days 3 and 4. Baseline burrowing performance was tested on day 5. Animals burrowing < 500 g in total were excluded from the experiment (<10%).

On day 14 post-surgery, the burrowing test was performed 1 h following drug(s) administration and burrowing performance was expresses as amount of gravel remaining in the tube (mean ± SEM).

### Nest construction

Nest building was assessed by placing rats into cages supplied with clean sawdust bedding. The nests were assessed according to a 5-point scale from 1 to 5 as follows: 1 = bedding not noticeably touched; 2 = bedding partially torn up; 3 = bedding mostly shredded but often no identifiable nest site; 4 = identifiable but flat nest; 5 = perfect or nearly perfect nest.

On day 14 post-surgery, CCI rats were treated with gabapentin and trazodone, and nest construction was assessed by placing rats into the nesting cages approximately 1 h before the dark phase and leaving them there until the next morning [17]. The results are expressed as mean ± SEM.

### Statistical analysis

An analysis of variance (ANOVA) followed by Dunnett’s test for multiple comparisons versus vehicle-treated group was performed for each experiment.

## Results

### Isobolographic analysis

**Fig 1:**
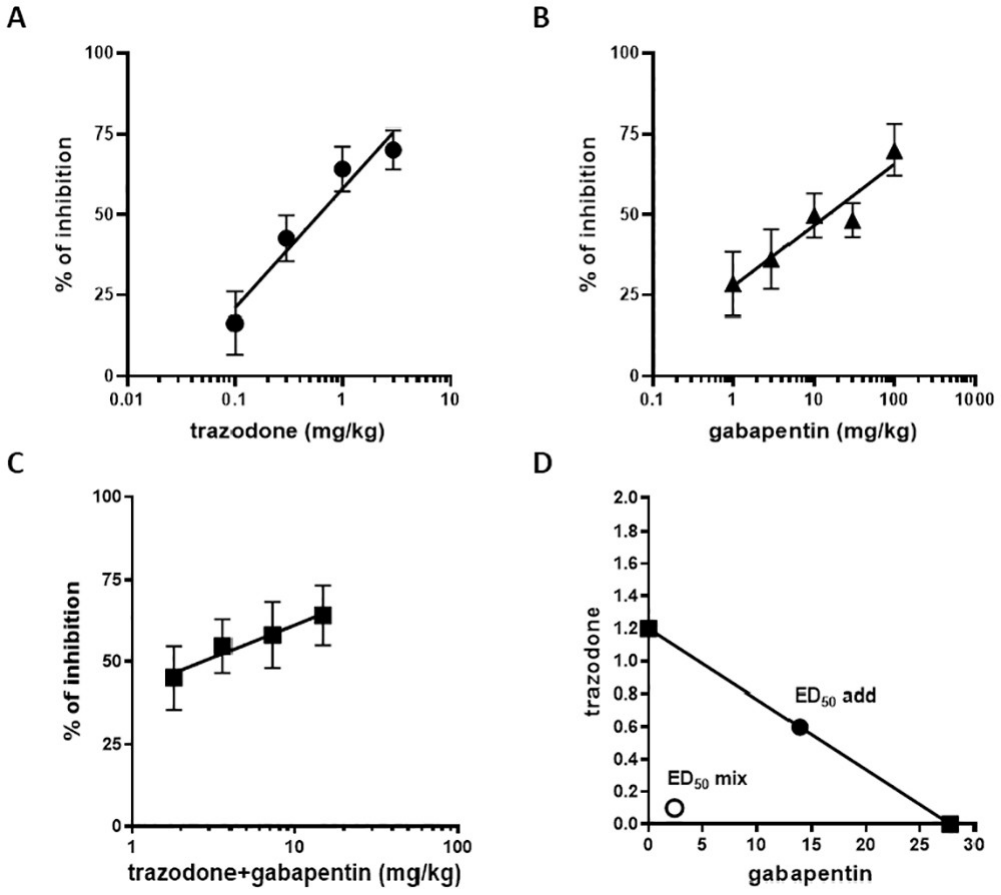
Antinociceptive effects of trazodone, gabapentin and trazodone and gabapentin combination in the writhing test in mice. Panels A, B and C: The results are expressed as percent inhibition of writhes induced by i.p. injection of acetic acid vs vehicle group. Individual drugs or the combination product were administered 1 h before acetic acid injection. Each point represents the mean ± SEM of percent inhibition of the number of writhes observed in 6 to 12 animals/group. Panel D: Isobologram for the combination of trazodone and gabapentin in the writhing test in mice. The ED_50_ values are individually plotted for each drug on the axes and the straight line connecting trazodone and gabapentin ED_50_ values is the theoretical additive line. Filled circles correspond to the theoretical additive ED_50_ and open circles correspond to the experimental ED_50_ of the combination product. n = 6–12 animals/group.

We used the writhing test in mice as a proof of principle assay to verify the hypothesis that trazodone and gabapentin have a synergistic antinociceptive effect when concomitantly administered. First, when administered individually in this acute pain model, trazodone (0.1–3 mg/kg, p.o.; Fig 1 panel A) and gabapentin (1–100 mg/kg, i.p.; Fig 1 panel B) caused a significant dose-dependent reduction in the number of writhes compared to the control group (F(4, 46) = 16.74, P < 0.0001 trazodone, F(5, 51) = 3.733, P = 0.0059 gabapentin), with ED_50_ values of 1.2 mg/kg (trazodone) and 27.8 mg/kg (gabapentin). In addition, taking into consideration the ratio between the trazodone and gabapentin ED_50_ values (1.2:27.8), the fixed drug-dose ratio, based on mass quantity for trazodone and gabapentin, was established at 1:23.2.

Following ED_50_ calculation, trazodone and gabapentin drug combination was administered in fixed-dose fractions of the calculated ED_50_ (1/2, 1/4, 1/8 and 1/16) determining an antinociceptive effect (Fig 1 panel C), with a significant reduction in the number of writhes (F(4, 33) = 6.885, P = 0.0004). These data allowed to calculate by linear regression analysis the ED_50_ of the combination product that resulted to be 2.5 mg/kg (made up of 0.1 mg/kg trazodone and 2.4 mg/kg gabapentin). An isobologram graph was then constructed by connecting the ED_50_ of trazodone plotted on the ordinate with the ED_50_ of gabapentin plotted on the abscissa to obtain the additivity line (Fig 1 panel D). According to the formula reported in the Method section, theoretical additive ED_50_ for the combination (ED_50 add_) was 14.5 mg/kg, representing 0.6 mg/kg trazodone plus 13.9 mg/kg gabapentin. Because the ED_50_ of the combination was 2.5 mg/kg, isobolographic analysis demonstrated that combined administration of trazodone and gabapentin has a synergistic antinociceptive effect, which was confirmed also by the calculation of the interaction index, which equaled to 0.17. In addition, it is worth to stress that no significant pharmacokinetic interactions were observed when the 2 drugs were co-administered (S1 Table).

### Chronic constriction injury in rats

#### Mechanical hyperalgesia

**Fig 2:**
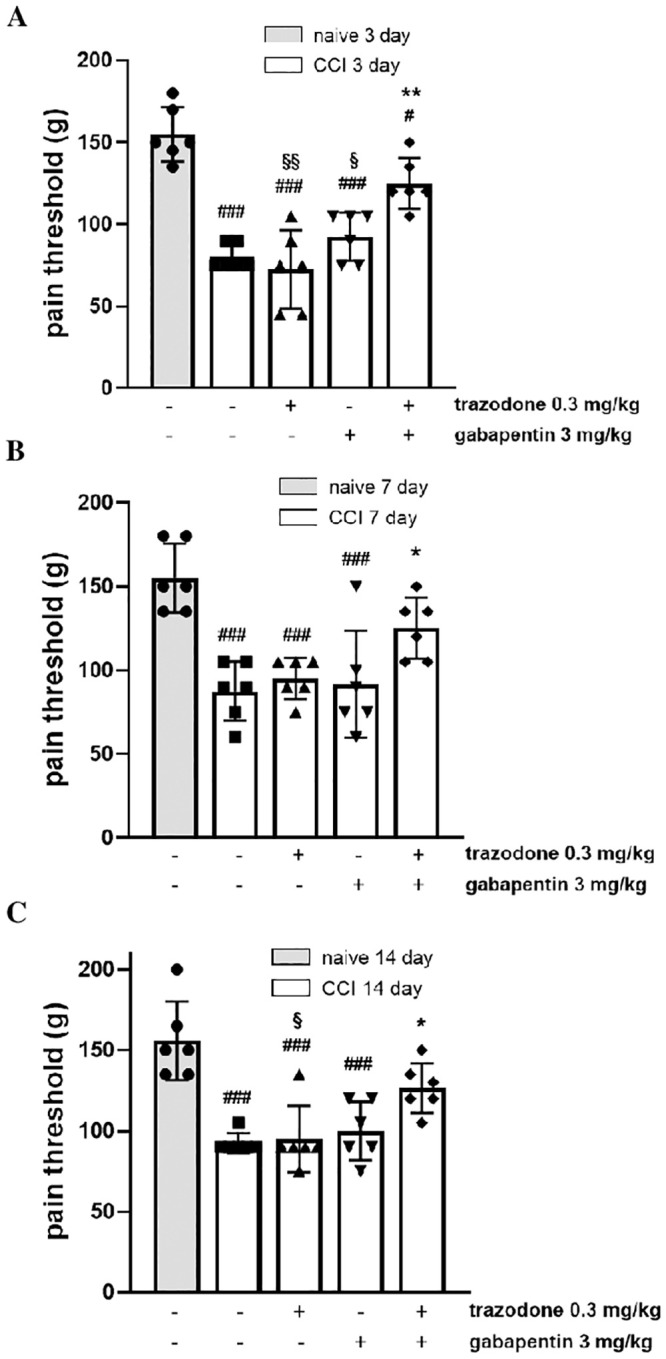
Effect of two ineffective doses of trazodone and gabapentin alone or in combination on mechanical hyperalgesia 3 (A), 7 (B) and 14 (C) days following rat sciatic nerve ligation. The results are expressed as pain threshold (in grams) recorded 1 h after treatment administration. ###P<0.001 vs naïve group; *P < 0.05, **P<0.01 vs vehicle CCI group; §P<0.05, §§P<0.01 vs trazodone-gabapentin CCI group. n = 6 animals/group.

The pain relieving effects of the combination were evaluated in the CCI rat model. This rat model of mononeuropathy causes mechanical hyperalgesia beginning approximately 3 days after nerve ligation and plateauing from day 7 [24,25]. In order to test for a synergistic effect, two trazodone and gabapentin doses, resulted ineffective in a previous dose-response study (S1 and S2 Figs), were administered alone and in combination. When CCI rats received trazodone 0.3 mg/kg or gabapentin 3 mg/kg alone, no antihyperalgesic effect was observed on any day (Fig 2). By contrast, a significant increase in the pain threshold was observed when drugs were administered at the same doses but in combination on days 3, 7 and 14 post surgery (Fig 2). Further analysis showed that co-administration of trazodone and gabapentin reached a %MPE value of 57.1±13.4 (day 3), 33.3±13.6 (day 7) and 36.4±15.0% (day 14) (F(11, 55) = 3.950, P = 0.0003).

### Weight bearing

**Fig 3:**
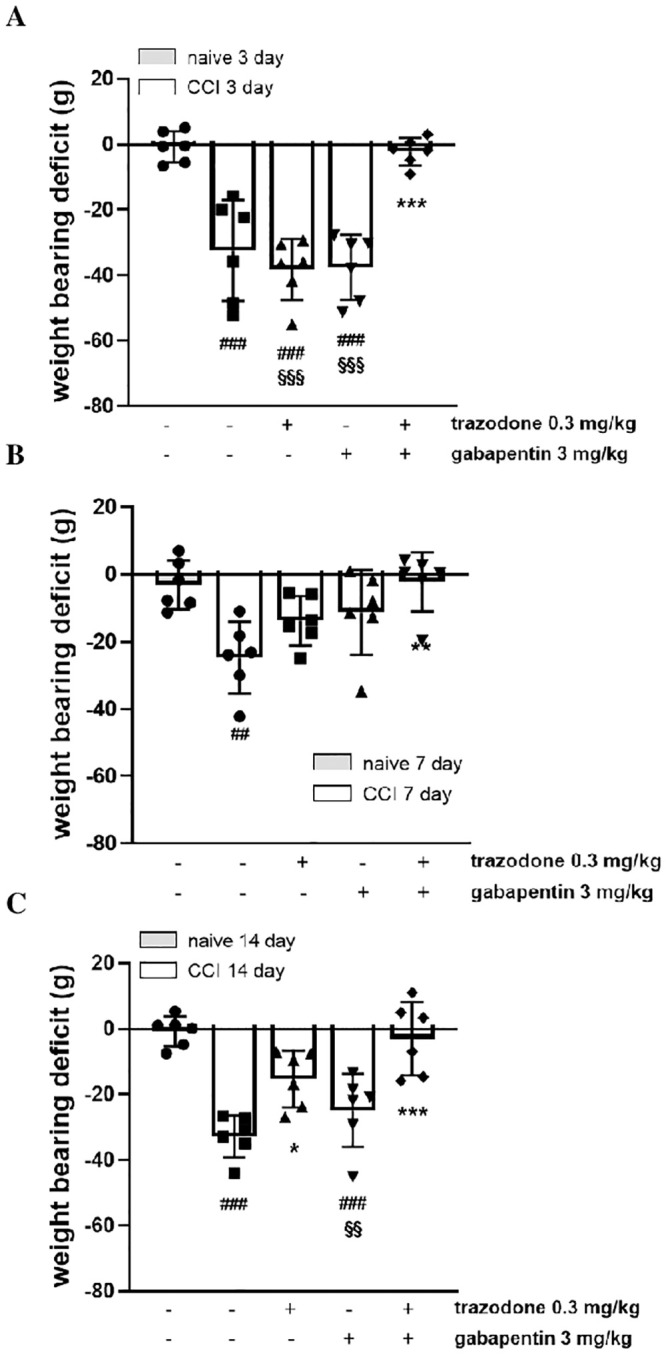
Effect of two ineffective doses of trazodone and gabapentin alone or in combination on weight bearing deficit 3 (A), 7 (B) and 14 (C) days following rat sciatic nerve ligation. The results are expressed as the difference between the contralateral and ipsilateral paws (in grams), calculated 1 h after treatment administration. ##P<0.1, ###P<0.001 vs naïve group; **P < 0.01, ***P < 0.001 vs vehicle CCI group; §§P<0.01, §§§P<0.001 vs trazodone-gabapentin CCI group. n = 6 animals/group.

Sciatic nerve ligation also impaired the ability of rats to bear weight in their hindlimbs. The difference between the weight borne on the contralateral paw and that borne on the ipsilateral paw was greater in the CCI + vehicle group (33.9 ± 5.4 g) than in the naïve + vehicle group (1.2 ± 3.1 g). As shown in Fig 3, when animals received the same combination treatment of the ineffective doses of trazodone (0.3 mg/kg) and gabapentin (3 mg/kg) used in the mechanical hyperalgesia assessment, a marked and significant inhibitory effect on CCI-induced weight-bearing deficit was observed on all tested days (F(11, 57) = 9.497, P < 0.0001).

### Burrowing and nest construction

**Fig 4:**
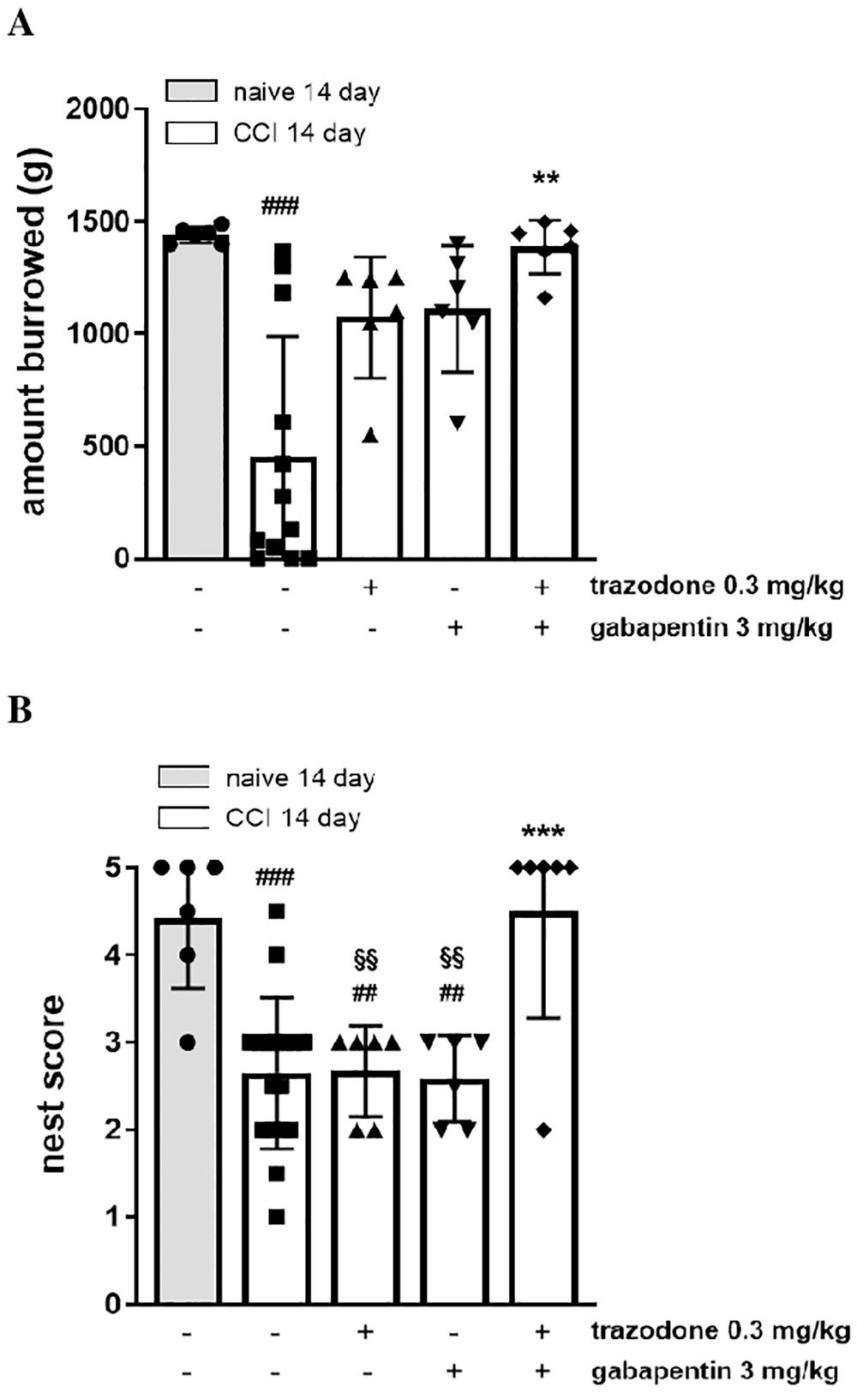
Effect of two ineffective doses of trazodone and gabapentin alone or in combination on the burrowing test (A) and the nest construction (B) 14 days following rat sciatic nerve ligation. The results are expressed as the amount of gravel displaced by burrowing and nest-building scores calculated 1 h after treatment administration. ##P<0.1, ###P<0.001 vs naïve group; **P < 0.01, ***P<0.001 vs vehicle CCI group; §§P<0.01 vs trazodone-gabapentin CCI group. n = 6–12 animals/group.

Burrowing is an innate rodent behaviour indicative of animal wellbeing and is therefore a useful preclinical measure of non-evoked pain [17,26,27]. We found that burrowing performance was significantly impaired in CCI rats only 14 days following sciatic nerve ligation (1340 ± 125 g naive + vehicle vs 701 ± 194 g CCI + vehicle). On day 14 post-surgery, the administration of the ineffective doses of trazodone (0.3 mg/kg) and gabapentin (3 mg/kg) used in the previous assays, did not restore burrowing performance, while the co-administration of the same doses but in combination significantly reversed burrowing deficit (Fig 4, panel A; F(4, 27) = 4.856, P = 0.0044).

Nest construction is common in rodent species and an increase in nest building behaviour seems to be indicative of animal wellbeing [17,18]. We found that nest construction was significantly impaired in CCI rats only 14 days following sciatic nerve ligation (4.4 ± 0.33 naive + vehicle vs 2.4 ± 0.24 CCI + vehicle). On day 14 post-surgery, 0.3 mg/kg trazodone or 3 mg/kg gabapentin individually did not restore nest construction behaviours, whereas the drugs co-administration significantly restored the ability of nestlet shredding (Fig 4, panel B; F(4, 36) = 10.09, P < 0.0001).

Taken together, our results seem to suggest that trazodone and gabapentin co-administration restores innate behaviors thus improving the global health and well-being state in CCI rats.

## Discussion

In the present study, we describe how the co-administration of low, ineffective doses of trazodone and gabapentin is active in reducing nociception both in an acute pain model in mice and in a chronic neuropathic assay in rats, in which a positive effect on the general well-being is also observed. Using the writhing test, which is an assay recommended for preliminary assessment of anti-nociceptive activity [28], we demonstrated through isobolographic analysis that trazodone and gabapentin exert a synergistic action on nociception and that ineffective doses of trazodone and gabapentin have a significant effect when they are co-administered. To explore the potential clinical relevance of these findings in chronic neuropathic pain conditions, we examined the effects of the trazodone and gabapentin combination in the CCI rat model. Our results show that the combination of low and ineffective doses of these two drugs exerts a significant antinociceptive effect on evoked nocifensive behaviors reflecting hypersensitivity to mechanical stimulations such as increasing pressure. Given that patients with neuropathic pain suffer a mixture of spontaneous and evoked pain symptoms, spontaneous pain-associated behaviours were also assessed in the CCI model using the weight-bearing distribution assessment [29]. On this parameter, whereas no data are available for trazodone, gabapentin is reported to exert a significant effect at high dosages (≥ 30 mg/kg) [30]. The results of our study show that the combination of ineffective doses of trazodone and gabapentin can reverse the weight-bearing deficit observed in CCI rats, confirming also on spontaneous pain symptoms, the effects assessed on evoked pain behaviors.

Pain and in particular chronic pain are recognized as conditions heavily affecting activities of daily living (ADL) in humans, a factor having an essential impact on quality of life in patients [31,32]. Nest building and burrowing are spontaneous behaviors that have been proposed to represent such ADL in rodents [17,33,34], and low performances in these home cage behaviors are considered indicative of abnormal behavioral functions or reduced well-being and therefore alternatives to simple reflexive responses [17,35–38]. The results obtained in the burrowing test and even more significantly in the nest construction assay in CCI animals suggest that the combination of trazodone and gabapentin can reinstate these spontaneous behaviors in a chronic pain model.

Although the mechanisms underlying the synergistic effect of trazodone and gabapentin have not been clarified in molecular terms, some hypotheses can be discussed based on the effects that are common to both drugs, the most evident being an indirect inhibition of glutamate release. Glutamate is the main excitatory neurotransmitter in the nervous system and it plays a key role in pain transmission from the periphery to the brain. Glutamate is also involved in central sensitization, which is associated with chronic pain. Glutamate action is mediated through ionotropic and metabotropic receptors that are involved in the fast synaptic response and the slow neuromodulatory response, respectively [39].

A recent paper has proposed that trazodone, as well as other 5-HT_2A_ antagonists, may act at single-nanomolar concentrations as indirect positive allosteric modulators of mGlu2/3 autoreceptors in the spinal cord glutamatergic terminals, where mGlu2/3 and 5-HT_2A_ receptors were shown to colocalize and interact in an antagonistic-like manner [40]. The consequence of this finding is that trazodone might be able to modulate glutamate exocytosis by indirectly activating release regulating receptors on glutamatergic nerve terminals. This observation is quite intriguing because elevated extracellular glutamate concentrations and excessive activation of NMDA glutamate receptors have been found in the spinal sensory synapses of animals with chronic constriction of the sciatic nerve [41] or receiving subcutaneous injection of an inflammatory agent such as formalin [42]. Furthermore, the blocking of extrasynaptic glutamate was shown to ameliorate the abnormal excitatory currents originating from NMDA receptor activation in neuropathic rats, whereas pharmacological blockade of glial glutamate transporters enhanced NMDA receptor activation in normal rats but not in neuropathic animals [43]. The overall data suggest that excessive extrasynaptic glutamate concentrations may be a key synaptic mechanism related to the enhanced activation of sensory neurons induced by nerve injury in the spinal dorsal horn. The restoration of glutamate homeostasis might therefore be a mechanism through which trazodone effectively interferes with the pathogenesis and development of neuropathic pain. Such hypothesis is also supported by our previous data showing that pre-treatment of CCI rats with the mGlu 2/3 receptor antagonist LY341495 significantly reduces the anti-hyperalgesic effects of trazodone [44].

Inhibition of K^+^-evoked glutamate release from rat neocortical and hippocampal slices by gabapentin has also been demonstrated and linked to the binding of gabapentin to the α2δ subunits of VSCCs [45,46]. Although some discordant data have been published [47,48], the capacity of gabapentin to modulate excitatory neurotransmitter release is considered a major mechanism to explain its efficacy on neuropathic pain both in preclinical and clinical settings. On such bases, we can conclude that indirect inhibition of glutamate release seems to be an effect common to both drugs, and it may well explain the synergistic antinociceptive efficacy of the trazodone and gabapentin combination observed in the CCI rat neuropathic pain model on different spontaneous and evoked behaviors.

Our results, showing that doses of trazodone and gabapentin normally ineffective can be combined to produce therapeutically relevant analgesia, are in line with such hypothesis and, even though they have yet to be confirmed in female animals [49], they are suggestive of an innovative clinical approach for the treatment of neuropathic pain. The development potential of the combination product is also reinforced by the observation that its activity is maintained after 14 days of continuous administration, making unlikely the insurgence of tolerance phenomena (S3 Fig); moreover, no serious adverse effects were reported in a randomized controlled pilot study with low doses of trazodone combined with gabapentin after 8 weeks of treatment in patients affected by painful diabetic neuropathy [50].

## Conclusion

Overall, the antinociceptive effects observed after administration of the trazodone and gabapentin combination in preclinical studies and the first reassuring safety data in humans lay the ground for the potential clinical application of the trazodone and gabapentin combination product in patients with chronic pain of neuropathic origin.

## Supporting information

S1 Fig Effect of trazodone oral administration on mechanical hyperalgesia 3 (A), 7 (B) and 14 (C) days following rat sciatic nerve ligation. The results are expressed as pain threshold (in grams) recorded 1 h after treatment administration. ##P<0.01, ###P<0.001 vs naïve group; *P < 0.05, **P<0.01, ***P<0.001 vs vehicle CCI group. n = 6 animals/group.
(TIF)

S2 Fig Effect of gabapentin intraperitoneal administration on mechanical hyperalgesia 3 (A), 7 (B) and 14 (C) days following rat sciatic nerve ligation. The results are expressed as pain threshold (in grams) recorded 1 h after treatment administration. ##P<0.01, ###P<0.001 vs naïve group; *P < 0.05 vs vehicle CCI group. n = 6 animals/group.
(TIF)

S3 Fig Effect of trazodone-gabapentin combination on mechanical hyperalgesia 3, 7 and 14 days following rat sciatic nerve ligation. The results are expressed as pain threshold (in grams) recorded 1 h after treatment administration. ###P<0.001 vs naïve group; *P < 0.05, ***P<0.001 vs vehicle CCI group. n = 6 animals/group.
(TIF)

S1 Table Pharmacokinetic parameters for trazodone and gabapentin. (PPTX)
