# Supplementary figures and images for: Synergistic interaction between trazodone and gabapentin in rodent models of neuropathic pain
Source: PLoS One. 2021 Jan 4;16(1):e0244649. doi: 10.1371/journal.pone.0244649 (PMC7781482; doi:10.1371/journal.pone.0244649)

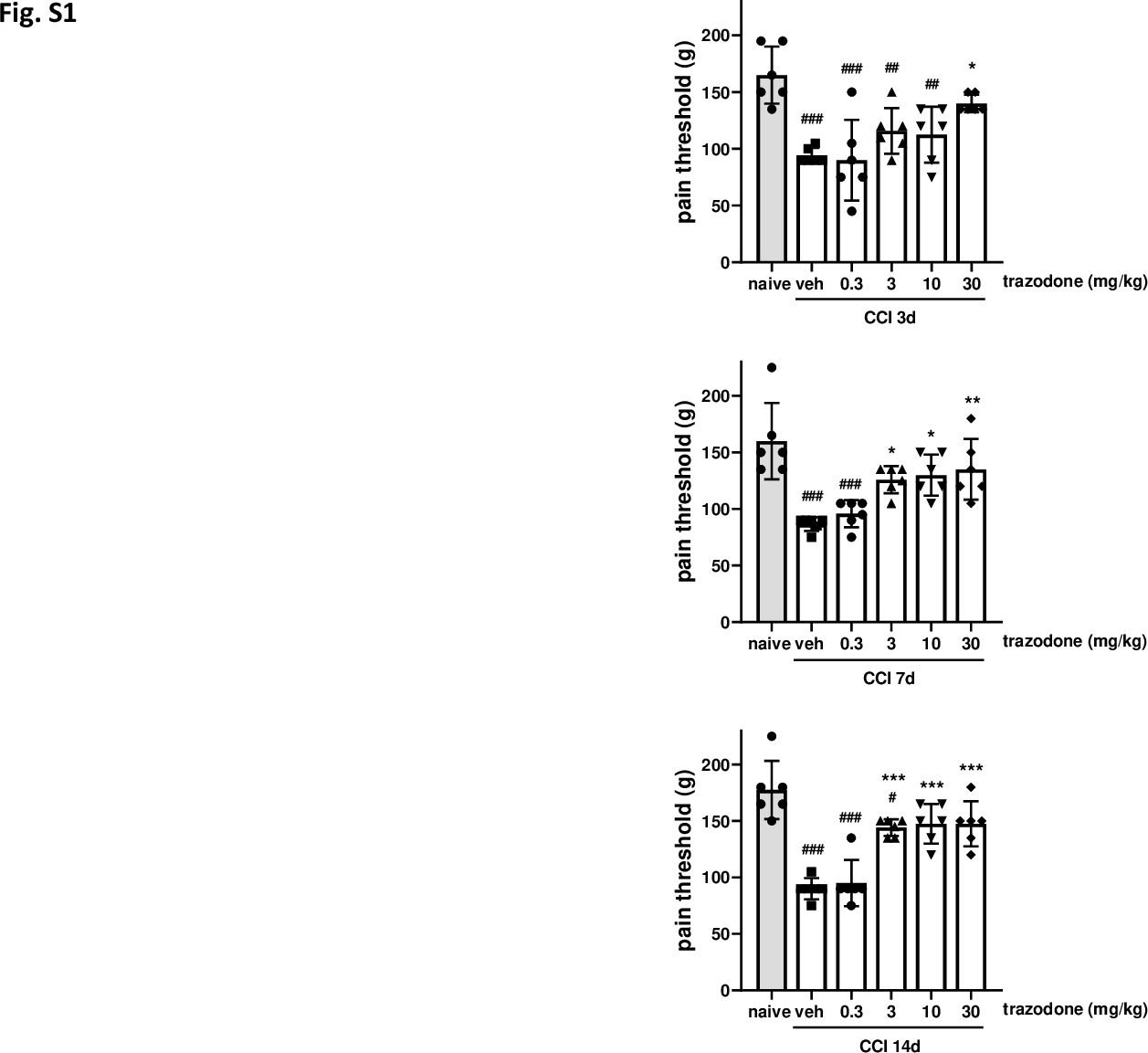

Supplement: S1 Fig — The results are expressed as pain threshold (in grams) recorded 1 h after treatment administration. ##P<0.01, ###P<0.001 vs naïve group; *P < 0.05, **P<0.01, ***P<0.001 vs vehicle CCI group. n = 6 animals/group. (TIF) [file pone.0244649.s001.tif]

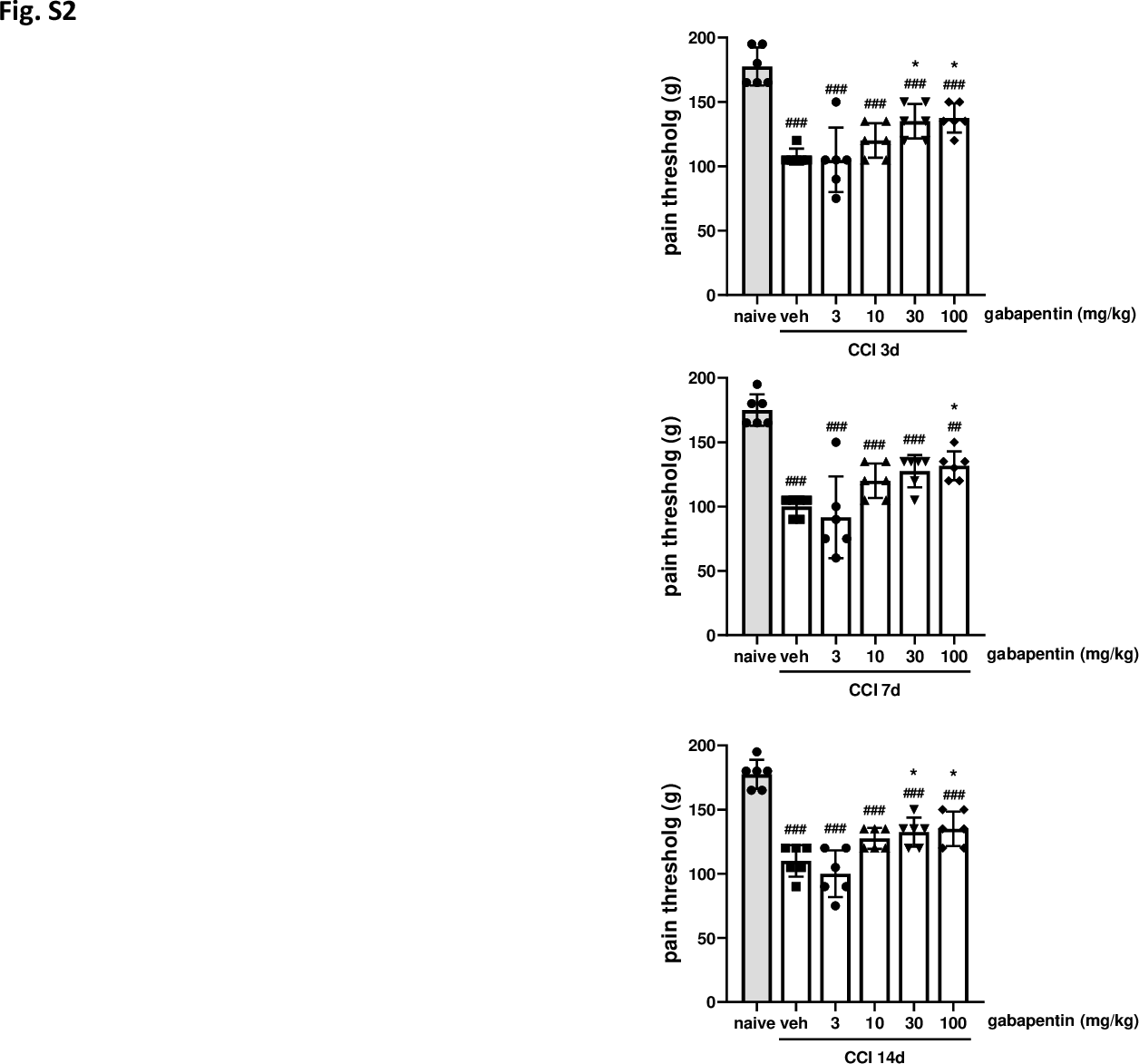

Supplement: S2 Fig — The results are expressed as pain threshold (in grams) recorded 1 h after treatment administration. ##P<0.01, ###P<0.001 vs naïve group; *P < 0.05 vs vehicle CCI group. n = 6 animals/group. (TIF) [file pone.0244649.s002.tif]

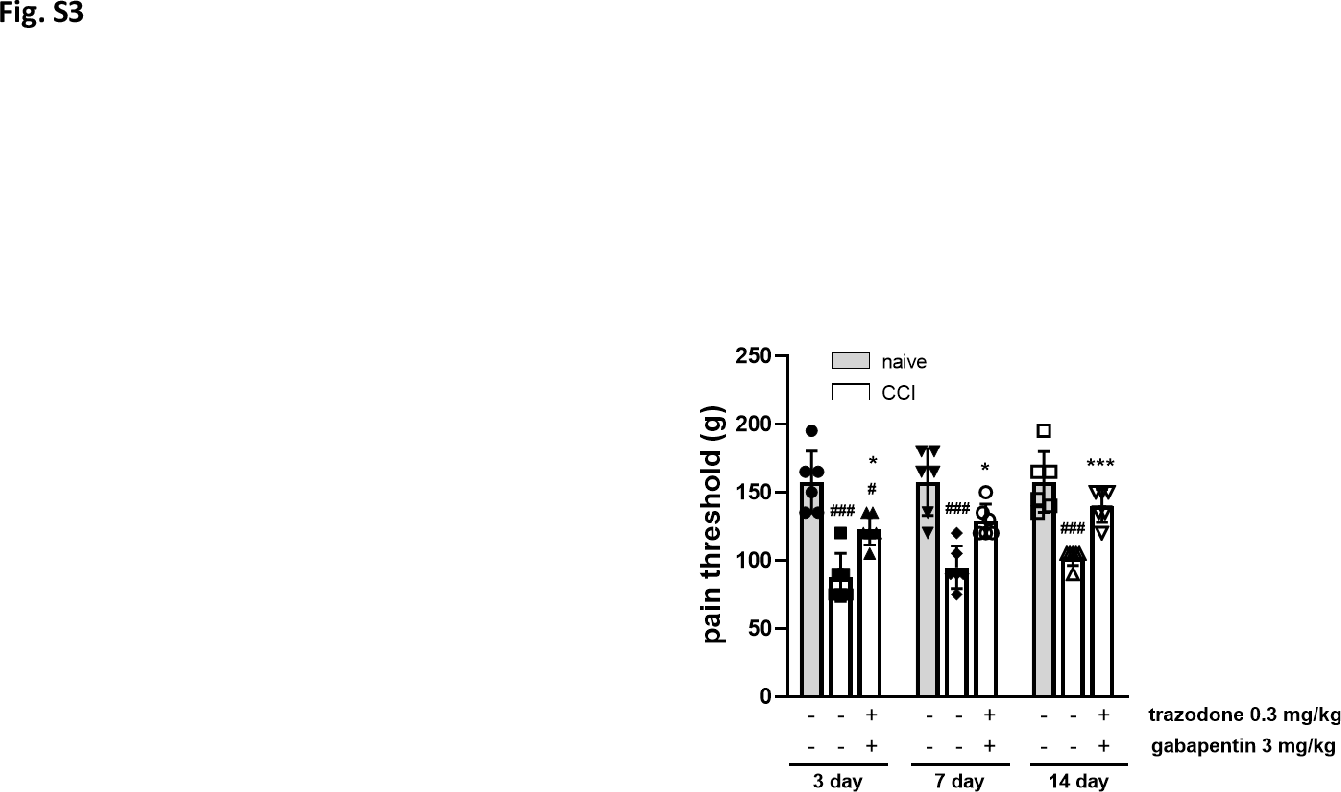

Supplement: S3 Fig — The results are expressed as pain threshold (in grams) recorded 1 h after treatment administration. ###P<0.001 vs naïve group; *P < 0.05, ***P<0.001 vs vehicle CCI group. n = 6 animals/group. (TIF) [file pone.0244649.s003.tif]
