# Supplementary material for: Synergistic interaction between trazodone and gabapentin in rodent models of neuropathic pain
Source: PLoS One. 2021 Jan 4;16(1):e0244649. doi: 10.1371/journal.pone.0244649 (PMC7781482; doi:10.1371/journal.pone.0244649)
Supplement: S1 Table — (PPTX) [file pone.0244649.s004.pptx]

## Slide 1
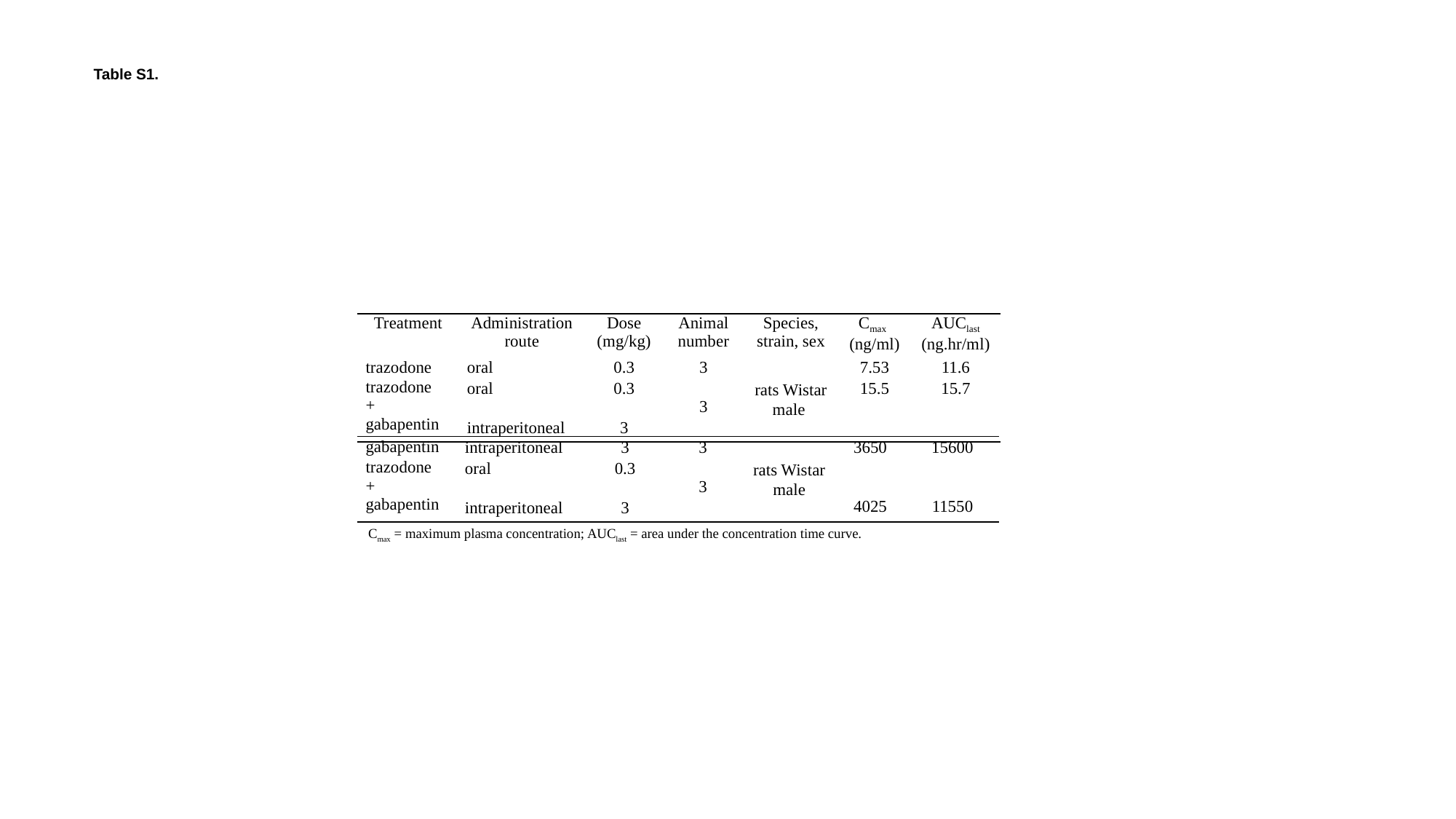

Table S1.
| Treatment | Administration route | Dose (mg/kg) | Animal number | Species, strain, sex | Cmax (ng/ml) | AUClast (ng.hr/ml) |
| --- | --- | --- | --- | --- | --- | --- |
| trazodone | oral | 0.3 | 3 | rats Wistar male | 7.53 | 11.6 |
| trazodone + gabapentin | oral   intraperitoneal | 0.3   3 | 3 | | 15.5 | 15.7 |
| gabapentin | intraperitoneal | 3 | 3 | rats Wistar male | 3650 | 15600 |
| --- | --- | --- | --- | --- | --- | --- |
| trazodone + gabapentin | oral   intraperitoneal | 0.3   3 | 3 | | 4025 | 11550 |
Cmax = maximum plasma concentration; AUClast = area under the concentration time curve.
